# Structural and functional coupling in cross-linking uracil-DNA glycosylase UDGX

**DOI:** 10.1042/BSR20231551

**Published:** 2024-01-09

**Authors:** Chuan Liang, Ye Yang, Ping Ning, Chenyan Chang, Weiguo Cao

**Affiliations:** Department of Genetics and Biochemistry, Clemson University, Room 049 Life Sciences Facility, 190 Collings Street, Clemson, SC 29634, U.S.A.

**Keywords:** DNA repair, mutagenesis, protein-DNA interactions, uracil, uracil-DNA glycosylase

## Abstract

Enzymes in uracil-DNA glycosylase (UDG) superfamily are involved in removal of deaminated nucleobases such as uracil, methylcytosine derivatives such as formylcytosine and carboxylcytosine, and other base damage in DNA repair. UDGX is the latest addition of a new class to the UDG superfamily with a sporadic distribution in bacteria. UDGX type enzymes have a distinct biochemical property of cross-linking itself to the resulting AP site after uracil removal. Built on previous biochemical and structural analyses, this work comprehensively investigated the kinetic and enzymatic properties of *Mycobacterium smegmatis* UDGX. Kinetics and mutational analyses, coupled with structural information, defined the roles of E52, D56, D59, F65 of motif 1, H178 of motif 2 and N91, K94, R107 and H109 of motif 3 play in uracil excision and cross-linking. More importantly, a series of quantitative analyses underscored the structural coupling through inter-motif and intra-motif interactions and subsequent functional coupling of the uracil excision and cross-linking reactions. A catalytic model is proposed, which underlies this catalytic feature unique to UDGX type enzymes. This study offers new insight on the catalytic mechanism of UDGX and provides a unique example of enzyme evolution.

## Introduction

Uracil generated from cytosine deamination is a common base damage in DNA. Enzymes in Uracil-DNA Glycosylase (UDG) superfamily are monofunctional glycosylases involved in removal of uracil and other types of base modifications from DNA. UDG superfamily is classified into at least six families based on the sequence conservation of three catalytic motifs. Family 1 UNG (uracil-*N*-glycosylase) is a group of narrow specificity but highly efficient enzymes, represented by *Escherichia coli* UNG as the first DNA glycosylase discovered [1–3]. Family 2 TDG/MUG is a group of UDGs with broad substrate specificity, excising uracil and other base lesions [4–11]. Human TDG, although previously discovered as a thymine DNA glycosylase removing thymine from G/T mispairs, is now recognized as a demethylase to remove formylcytosine (fC) and carboxylcytosine (caC) generated by TET-mediated oxidation of methylcytosine (mC) during the demethylation process [12,13]. Family 3 SMUG1 contains several subfamilies with different substrate specificities [14–20]. Family 4 UDGa was found as a group of narrow specificity but highly efficient enzymes in prokaryotic organisms [21–24]. Family 5 UDGb enzymes were found in bacteria and archaea with relatively broad substrate specificities and moderate catalytic efficiencies [25–27]. Family 6 HDG is predominantly a hypoxanthine DNA glycosylase [28].

In recent years, a group of highly unusual UDG (UDGX) was found in certain bacterial species, in which the enzyme forms a covalent bond with an AP site after excision of a uracil base, i.e., the enzyme and the DNA become a cross-linked protein–DNA complex after the first catalytic step to remove a uracil [29]. This unique enzymatic property has been explored to develop tools for detection or visualization of uracil in DNA [30–32]. Structural and biochemical studies have identified a histidine residue (H109) in the extended loop of motif 3 in UDGX as the site of cross-linking [33,34]. This extended loop has been recognized as a significant structural feature different from the other families in UDG superfamily. Despite of the availability of crystal structures of UDGX, the basic kinetic information is missing and the roles of some key residues in uracil excision and AP site cross-linking are not well defined. The unique enzymatic function of UDGX in relationship to its catalytic motifs as well as structure is not completely understood. In this work, a combination of quantitative and biochemical studies was conducted to define the roles of a series of amino acid residues of motifs 1, 2 and 3 in uracil excision and protein–DNA cross-linking. Furthermore, a catalytic mechanism underlying the unique enzymatic properties of UDGX enzymes was proposed.

## Results

**Figure 1:**
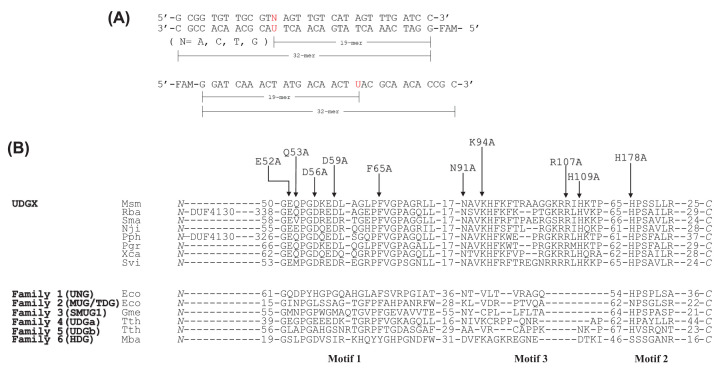
Substrates and sequence alignment (**A**) Sequences of uracil-containing DNA substrates (A/U, C/U, T/U and G/U base pair containing double-stranded DNA and single-stranded U DNA). (**B**) Sequence alignment of UDGX enzymes with other family enzymes in UDG superfamily. GenBank accession numbers are shown after the species names. UDGX: Msm, *Mycobacterium smegmatis*, WP_011726794.1; Rba, *Rhodopirellula baltica*, WP_011119624.1; Sma, *Saccharomonospora marina*, WP_009153389.1; Nji, *Nonomuraea jiangxiensis*, WP_090946880.1; Pph, *Paraburkholderia phytofirmans*, WP_012433335.1; Pgr, *Paraburkholderia graminis* C4D1M, EDT13144.1; Xca, *Xanthomonas campestris*, WP_011038956.1; Svi, *Saccharomonospora viridis*, WP_015785655.1. Family 1 (UNG): Eco, *Escherichia coli* O157:H7 str. EDL933, NP_289138.1; Family 2 (MUG/TDG): Eco, *Escherichia coli*, P0A9H1.1; Family 3 (SMUG1): Gme, *Geobacter metallireducens* GS-15, YP_383069.1; Family 4 (UDGa): Tth, *Thermus thermophilus*, WP_011228142.1; Family 5 (UDGb): Tth, *Thermus thermophilus*, WP_011173217.1; Family 6 (HDG): Mba, *Methanosarcina barkeri*, WP_011305765.1.

UDGX was first discovered in *Mycobacterium smegmatis* [29]. We searched sequenced genomes for the existence of *udgx* genes in other species. So far, the *udgx* genes were only detected in bacteria, no *udgx* genes were found in archaea or eukaryotic organisms (Figure 1B and Supplementary Figure S1). To gain a better understanding of the distribution of *udgx* genes in bacteria, we superimposed *udgx* genes to a bacterial tree of life. Within the 81 bacterial species shown in Supplementary Figure S1, we found limited and scattered distribution in a variety of bacteria ranging from *Mycobacterium smegmatis* in actinobacteria to *Erythrobacter litoralis* in alphaproteobacteria to *Caballeronia arationis* in betaproteobacteria. Interestingly, some genomes contain more than one *udgx* genes within a family. For example, *Rhizobium leguminosarum* contains three *udgx* genes in its genome and there is a cluster of betaproteobacteria with multiple *udgx* genes in their genomes (Supplementary Figure S1). UDGX from *Mycobacterium smegmatis* has served as a prototype to elucidate the unique biochemistry and catalytic mechanism of UDGX enzymes.

**Figure 2:**
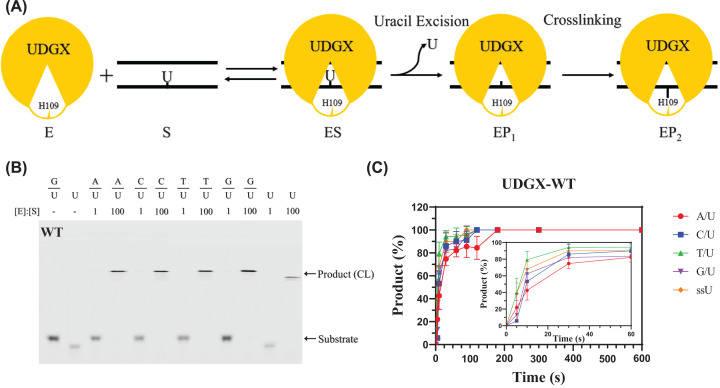
Catalytic scheme and biochemical analyses of UDGX (**A**) Catalytic scheme of UDGX on U-containing DNA substrate. E: Enzyme; S: Substrate; ES: Enzyme–Substrate complex; EP_1_: Complex of enzyme and uracil excised DNA; EP_2_: DNA-UDGX cross-linking complex. (**B**) Cross-linking analysis of wild-type UDGX. DNA cross-linking assays were performed as described in Materials and methods. Product (CL) indicates DNA-UDGX cross-linking product. (**C**) Time-course analysis of wild-type UDGX on U-containing DNA substrates. (●) A/U; (■) C/U; (▲) T/U; (▼) G/U; (♦) single-stranded U (ssU). The assays were performed as described in Materials and methods under Enzyme kinetics analysis with modification. Five types of uracil DNA substrates (20 nM) were incubated with 1000 nM wild-type UDGX enzyme, and samples were collected at 5, 10, 30 s, 1, 1.5, 2, 3, 5 and 10 min. Data are shown as the average of three independent experiments and the standard deviation for each point is shown.

A general reaction scheme of UDGX is shown in Figure 2A. Like other UDG enzymes, UDGX searches for uracil in DNA and forms an ES complex and then removes uracil base from DNA and leaves an AP site (Figure 2A, uracil excision step). Unlike conventional UDG enzymes, histidine 109 of motif 3 in UDGX forms a covalently cross-linked product with the remaining AP site (Figure 2A, cross-linking step). A representative SDS-PAGE analysis of UDGX-catalyzed reaction is shown in Figure 2B. Under the assay conditions in which the enzyme:substrate (E:S) ratio was 1:1, we did not detect any cross-linked product (bands of DNA–protein complex with higher molecular weight in the gel). When the E:S ratio was 100:1, we observed complete conversion of substrate (bands of oligodeoxynucleotides with lower molecular weight in the gel) to the cross-linked product for all basepairs (A/U, C/U, T/U, G/U) and single-stranded uracil-containing DNA (Figure 2B). A time-course analysis of the uracil excision and following AP site cross-linking reactions catalyzed by UDGX with all five substrates (E:S = 50:1) was performed to show the reaction efficiencies (Figure 2C). Within 60 s, over 80% of the reactions were completed (Figure 2C, inset) and within 180 s, all the substrates were converted to cross-linked products (Figure 2C). The reaction with the A/U base pair was slower than other substrates, consistent with the notion that A/U forms a normal Watson–Crick base pair while C/U, T/U and G/U form mismatch base pairs. To more quantitatively characterize the kinetic behavior of the UDGX enzyme, we measured the kinetic parameters using a previously established approach [24,35,36]. Because the *K*_m_ was too large, it was unable to determine the *K*_m_ values alone (Supplementary Figure S2), instead, we obtained *k*_2_/*K*_m_ values for G/U and A/U basepairs (Table 1). Consistent with the time-course analysis shown in Figure 2C, the reaction with A/U was 2-fold slower than with G/U as judged by the *k*_2_/*K*_m_ values.

**Table 1:** Kinetic parameters of UDGX on G/U and A/U base pairs*

Enzyme	G/U			A/U		
	*K*_m_ (M)	*k*_2_ (s^−1^)	*k*_2_/*K*_m_ (M^−1^s^−1^)	*K*_m_ (M)	*k*_2_ (s^−1^)	*k*_2_/*K*_m_ (M^−1^s^−1^)
Wild-Type	N.D. ^†^	N.D.	1.4 (0.09) × 10^4^	N.D.	N.D.	7.4 (1.8) × 10^3^
E52A	2.2 (0.4) × 10^−6^	2.8 (0.1) × 10^−4^	1.3 (0.2) × 10^2^	1.1 (0.2) × 10^−6^	1.6 (0.2) × 10^−4^	1.5 (0.1) × 10^2^
Q53A	N.D.	N.D.	8.4 (1.2) × 10^3^	N.D.	N.D.	8.1 (1.4) × 10^3^
D56A	N.D.	N.D.	6.0 (2.0) × 10^3^	2.4 (1.2) × 10^−6^	4.5 (1.3) × 10^−3^	2.0 (0.3) × 10^3^
D59A	N.A.^‡^	N.A.	N.A.	N.A.	N.A.	N.A.
F65A	N.A.	N.A.	N.A.	N.A.	N.A.	N.A.
N91A	N.A.	N.A.	N.A.	N.A.	N.A.	N.A.
K94A	N.A.	N.A.	N.A.	N.A.	N.A.	N.A.
R107A	N.A.	N.A.	N.A.	N.A.	N.A.	N.A.
H109A^§^	3.9 (1.0) × 10^−7^	6.4 (0.7) × 10^−4^	1.7 (0.3) × 10^3^	2.3 (0.9) × 10^−7^	1.8 (0.04) × 10^−4^	8.8 (3.6) × 10^2^
H178A	N.A.	N.A.	N.A.	N.A.	N.A.	N.A.
Q53A-H109A^§^	N.D.	N.D.	2.6 (0.3) × 10^3^	N.D.	N.D.	1.5 (0.1) × 10^3^
E52A-H109A	N.A.	N.A.	N.A.	N.A.	N.A.	N.A.

* Enzyme kinetics analysis was performed as described in Materials and methods. For the Q53A-H109A mutant on A/U DNA substrate, samples were collected at 10, 20, 30, 40, 55, 70, 85 and 100 min due to the slow reaction rate. Data are shown as the average of three independent experiments. S.D. values are shown in parenthesis.

† N.D. Not determined due to large *K_m_*.

‡ N.A. No activity detected under assay condition.

§ For H109A and Q53A-H109A mutants, *k_2_* values reflect uracil excision only, whereas *k_2_* values for wild-type and the other mutants reflect uracil excision and subsequent cross-linking process together.

A comparison of motifs 1, 2 and 3 between representative UDGX enzymes with UDGs from other families is shown in Figure 1B. UDGX is most closely related to family 4 UDGa. For example, Msm UDGX shares 37.20% sequence identity with *Thermus thermophilus* UDGa. Some sequence conservation within the motifs is also evident, as most UDGX enzymes contain GEQP, N and HP at the start of each motif while most of family 4 UDGa enzymes contain GEGP, N and HP, respectively (Figure 1B). Despite of these similarities, notable differences exist between UDGX and family 4 UDGa, especially in motif 1 and motif 3. In addition to the extra R-loop at the end of motif 3, UDGX has two conserved negatively charged aspartate residues (D56, D59) in motif 1 and a conserved positively charged residue (R107) in motif 3, which are missing in other UDG families (Figure 1B). Another residue, K94, is also highly conserved in UDGX enzymes, which is not observed in other UDG families except for family 4 UDGa.

**Figure 3:**
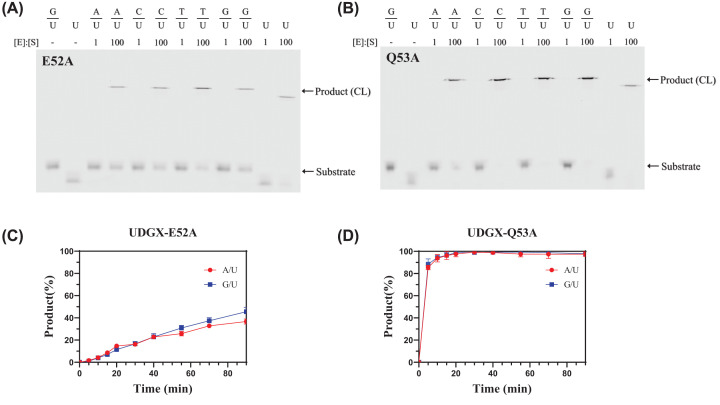
DNA cross-linking analysis of UDGX-E52A and Q53A mutants DNA cross-linking assays were performed as described in Materials and methods. Product (CL) indicates DNA-UDGX cross-linking product. Time-course analysis assays were performed as described in Materials and methods under Enzyme kinetics analysis with modification. Uracil DNA substrates were incubated with 1000 nM of UDGX-E52A or Q53A mutant. Data are shown as the average of three independent experiments and the standard deviation for each point is shown. (**A**) Cross-linking analysis of UDGX-E52A mutant. (**B**) Cross-linking analysis of UDGX-Q53A mutant. (**C**) Time-course analysis of UDGX-E52A mutant. (●) G/U; (■) A/U. (**D**) Time-course analysis of UDGX-Q53A mutant. (●) G/U; (■) A/U.

Based on the sequence alignment and previous structural studies, we chose ten sites among the three motifs for mutational studies and constructed ten single mutants (Figure 1B). As with the wild-type enzyme, the mutant proteins were subject to DNA cross-linking assay first to test their ability to form a covalent bond after uracil excision. At E52 and Q53 positions, we constructed E52A and Q53A mutants. As shown in Figure 3, E52A and Q53A still retained their ability to cross-link to the AP site after uracil excision with all five substrates (A/U, C/U, T/U, G/U and single-stranded U) as cross-linking product observed in the SDS-PAGE gel. The time-course analyses of the cross-linking reactions with A/U and G/U base pairs indicated that both E52A and Q53A mutants, especially the former, were less efficient in forming the cross-linked product in comparison with the wild-type UDGX enzyme (Figures 2 and 3). To further quantitatively define the kinetic properties, we measured the *K*_m_, *k*_2_ and *k*_2_/*K*_m_ values. E52A showed a *K*_m_ value in the micromolar range for both G/U and A/U base pairs and *k*_2_ of 2.8 × 10^−4^/s for G/U and 1.6 × 10^−4^/s for A/U (Table 1). Judging by the *k*_2_/*K*_m_ values, E52A was two-orders of magnitude slower than the wild-type enzyme for the G/U base pair and one-order of magnitude for the A/U base pair (Table 1). The kinetic behavior of Q53A was similar to the wild-type enzyme but the catalytic efficiency was about 2-fold lower for the G/U and comparable for the A/U (Table 1).

**Figure 4:**
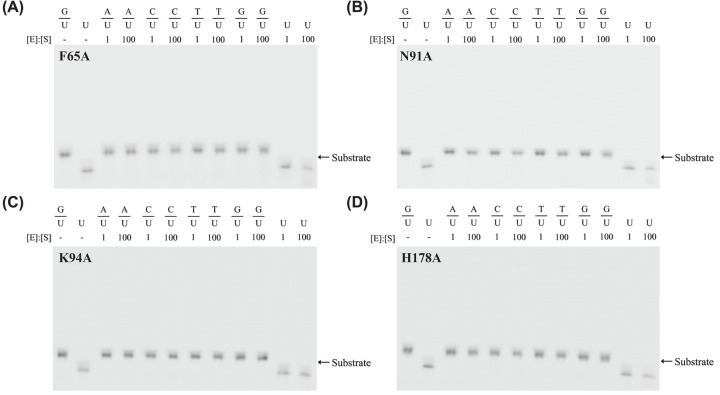
DNA cross-linking analysis of UDGX-F65A, N91A, K94A and H178A mutants DNA cross-linking assays were performed as described in Materials and methods. (**A**) UDGX-F65A mutant. (**B**) UDGX-N91A mutant. (**C**) UDGX-K94A mutant. (**D**) UDGX-H178A mutant.

F65 position is highly conserved in UDG enzymes being either phenylalanine or tyrosine. In UDG structures, the aromatic sidechain from Phe or Tyr stacks on the uracil base. Consistent with previous studies, F65A lost its catalytic activity on all substrates tested due to the loss of the aromatic ring in the amino acid sidechain since no cross-linking product was detected in the SDS-PAGE gel (Figure 4A). Mutations at N91 position (N91A) and K94 position (K94A) in motif 3 inactivated the enzyme, resulting in loss of cross-linking activity on uracil DNA (Figure 4B,C). The first residue in motif 2, H178, is known as an important catalytic residue in some families of UDG enzymes. As expected, H178A mutant completely lost its catalytic activity on all the uracil-containing DNA substrates tested (Figure 4D). Uracil excision assays also confirmed the loss of uracil excision activity of these four mutants. To investigate if these mutations affect the folding of the proteins, thermal shift protein stability assays were performed. Results showed that N91A and H178A mutants still had similar folding to the wild-type UDGX (Supplementary Figure S3A), however, F65A and K94A mutants were unfolded (Supplementary Figure S3B).

**Figure 5:**
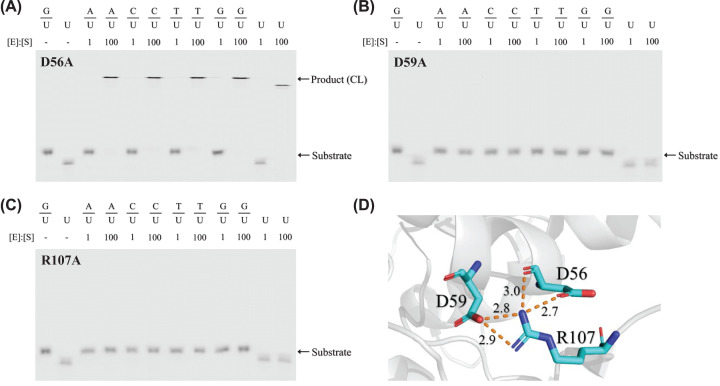
Tripartite interactions by D56, D59 and R107 DNA cross-linking assays were performed as described in Materials and methods. (**A**) Cross-linking analysis of UDGX-D56A mutant. Product (CL) indicates DNA-UDGX cross-linking product. (**B**) Cross-linking analysis of UDGX-D59A mutant. (**C**) Cross-linking analysis of UDGX-R107A mutant. (**D**) Close-up view of interactions by D56, D59 and R107 in crystal structure (PDB code: 6IO9 [37]). Distances are shown in Å.

Previous structural analysis indicated the possibility of salt bridges among D56 and D59 of motif 1 and R107 of motif 3 in UDGX [37]. To definitively determine the interactions among the three residues, we investigated the catalytic activities of D56A, D59A and R107A mutants. D56A exhibited cross-linking activity with all five substrates (A/U, C/U, T/U. G/U and single-stranded U) (Figure 5A). Judging by the *k*_2_/*K*_m_ values from the kinetics analyses, D56A is 2-fold slower for the G/U basepair and approximately 4-fold slower for the A/U base pair than the wild-type (Table 1). D59A and R107A completely lost their catalytic activities with neither observation of cross-linking product in the SDS-PAGE gel nor truncated DNA product detected in uracil excision assays (Figure 5B,C and Table 1), furthermore, the protein folding of these two mutants were severely affected (Supplementary Figure S3B), suggesting that D59 and R107 play an essential role in establishing the stable structural interactions between motif 1 and motif 3. Structurally, the guanidino sidechain of R107 of motif 3 forms bidentate salt bridges with the carboxyl sidechain of D59 of motif 1 within 3 Å distance and interacts with D56 through a hydrogen bond with the mainchain amide group and the carboxyl sidechain through a salt bridge (Figure 5D).

**Figure 6:**
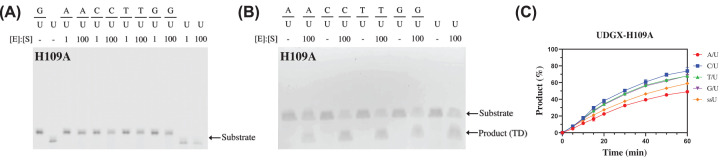
DNA cross-linking and uracil excision analyses of UDGX-H109A mutant (**A**) DNA cross-linking analysis of UDGX-H109A mutant. Assays were performed as described in Materials and methods. (**B**) Uracil excision analysis of UDGX-H109A mutant. Assays were performed as described in Materials and methods but visualized by electrophoresis on a 15% urea denaturing polyacrylamide gel. Product (TD) indicates truncated DNA product after NaOH/heat treatment. (**C**) Time course analysis of UDGX-H109A mutant. (●) A/U; (■) C/U; (▲) T/U; (▼) G/U; (♦) single-stranded U (ssU). The assays were performed as described in Materials and methods under enzyme kinetics analysis with modification. Uracil DNA substrates were incubated with 1000 nM UDGX-H109A enzyme. Samples were collected at 5, 10, 15, 20, 30, 40, 50 and 60 min. Data are shown as the average of three independent experiments and the standard deviation for each point is shown.

H109 of motif 3 has been identified as the site of cross-linking and substitution at this position renders the enzyme inactive in cross-linking [29,33,37,38]. Consistent with previous reports [29,38], though H109A did not exhibit cross-linking activity in SDS-PAGE analysis (Figure 6A), it still showed uracil excision activity as illustrated by urea-denaturing electrophoresis analysis on all five substrates tested (A/U, C/U, T/U, G/U, single-stranded U) (Figure 6B). A time-course analysis of the uracil excision reaction catalyzed by H109A mutant indicated that the uracil excision efficiency was much lower than the wild-type UDGX with the activity on A/U as the lowest (Figure 6C). Kinetics analyses showed that the uracil excision by H109A was one order of magnitude slower than the cross-linking by the wild-type UDGX for the G/U and A/U base pairs, respectively, as judged by the *k*_2_/*K*_m_ values (Table 1). When H109A mutation was coupled with E52A mutation, although the double mutant still maintained the normal folding (Supplementary Figure S3A), E52A-H109A totally lost its catalytic activity (Table 1), which is consistent with a previous study showing significantly decreased activity of a E52N-H109S double mutant [38].

**Figure 7:**
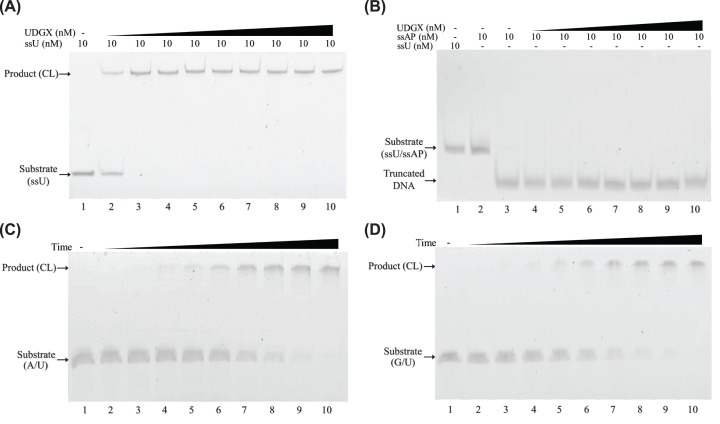
Uracil excision and AP site cross-linking coupling of UDGX (**A**) Cross-linking analysis of UDGX on single-stranded U DNA substrate (ssU). The assays were performed as described in Materials and methods under DNA cross-linking assay with following modifications. Approximately 10 nM of ssU DNA substrates were incubated with increasing concentration (ranging from 10 to 1000 nM) of UDGX at 37°C for 60 min. Samples were electrophoresed for 85 min on a 15% urea denaturing polyacrylamide gel and visualized by fluorescence scanning. Product (CL) indicates DNA-UDGX cross-linking product. (**B**) Cross-linking analysis of UDGX on single-stranded AP site DNA substrate (ssAP). The assays were performed similarly as described for the assays on ssU substrates in Figure 7A. Except for samples in lanes 1 and 2, samples in lanes 3–10 were treated with NaOH and heat. The AP sites not cross-linked by UDGX were cleaved and the truncated DNA products are shown in the gel. (**C**) Cross-linking analysis of UDGX on A/U base pair DNA substrate. The assays were performed as described in Materials and Methods under enzyme kinetics analysis with 500 nM of UDGX. The assays were stopped by adding 10 μl of 100 mM NaOH at 10, 30 s, 1, 1.5, 2.5, 5, 7.5, 10 and 15 min. (**D**) Cross-linking analysis of UDGX on G/U base pair DNA substrate. The assays were performed as described in Materials and Methods under enzyme kinetics analysis with 500 nM of UDGX. The assays were stopped by adding 10 μl of 100 mM NaOH at 10, 30 s, 1, 1.5, 2.5, 5, 7.5, 10 and 15 min.

A hallmark of UDGX is its ability to cross-link to the AP site generated after uracil excision. To understand how UDGX reacts with an AP site, we prepared an AP site substrate after removal of uracil in single-stranded oligodeoxynucleotides by a conventional UDG enzyme. Albeit UDGX was able to convert a single-stranded uracil-containing DNA substrate to the cross-linked product (Figure 7A), it was incapable of converting the AP site substrate to a cross-linked product even at the highest enzyme concentration tested (Figure 7B). Furthermore, no truncated DNA fragment (as a result of forming AP site) was detected with the A/U (Figure 7C) or the G/U (Figure 7D) substrates in 15-min incubation with the UDGX enzyme. The implication of this finding will be discussed in detail later.

## Discussion

Since the discovery of the first DNA glycosylase, family 1 UNG, in *E. coli* in 1974, UDG enzymes have been discovered in many bacteria, archaea and eukaryotes. Almost all living organisms possess at least one UDG enzyme in their genomes and many have multiple, forming a UDG superfamily with families of a variety of catalytic functions, going far beyond as a narrow specificity family 1 UNG-type DNA repair enzyme. Unlike many other enzyme superfamilies, a rather unique feature of UDG superfamily is that none of the sites is completely conserved in all UDG enzymes [39], although sequence or motif conservation is evident within UDG families. This phenomenon suggests a broad catalytic diversity in UDG superfamily. UDGX represents a class of UDG enzymes that not only possess uracil excision ability but also acquired an unprecedented cross-linking function upon uracil removal. Taking advantage of the solved crystal structures and building on previous studies, this work presents a comprehensive biochemical and enzymological analysis of UDGX and provides catalytic insights to understand the inner working of this bi-functional enzyme.

### Structural coupling

**Figure 8:**
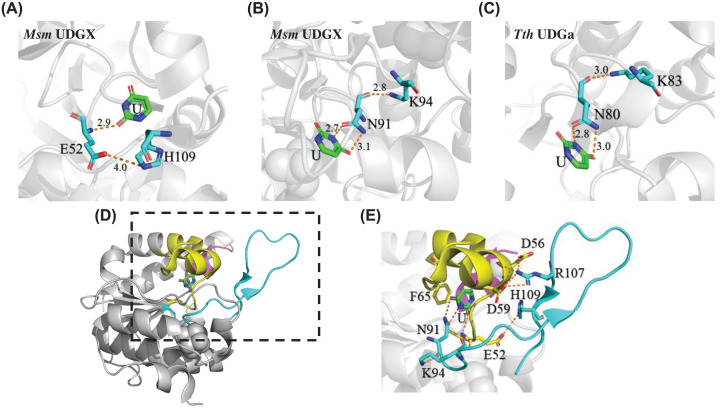
Inter-motif and intra-motif interactions in UDGX structure (**A**) Interaction between E52 of motif 1 and H109 of motif 3 of UDGX (PDB code: 6IOA [37]) (**B**) Interaction between N91 and K94 of motif 3 of UDGX (PDB code: 6IOA [37]). (**C**) Interaction between N80 and K83 of motif 3 of Tth UDGa (PDB code: 1UI0 [21]). Distances between residues were shown in Å. (**D**) Relative positions of motifs 1, 2 and 3 in UDGX. Motifs 1, 2 and 3 were highlighted by yellow, magenta and cyan, respectively (PDB code: 6IOA [37]). (**E**) Close-up view of relative positions of motif 1 and 3 driven by residue interactions (dashed rectangle area in Figure 8D).

**Figure 9:**
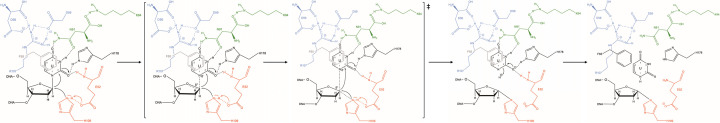
Proposed catalytic mechanism of uracil excision and AP site cross-linking by UDGX Supported by the inter-motif interactions between E52 and H109, D56–D59–R107 tripartite interactions and intra-motif interation between K94 and N91 (the interactions by D56, D59 and R107 are highlighted in blue; K94–N91 interaction is highlighted in green; and the E52–H109 interaction is highlighted in red), E52 and H178 interact with O2 of uracil, and N91 forms bidentate interactions with O3 and N4 of uracil, promoting the breakage of N-glycosidic bond and the departure of uracil, meanwhile, H109 initiates attack at the C1′ position of the deoxyribose to form a new covalent C-N bond and to further push the departure of the uracil base, as shown in the transition states (square brackets with ‡). After the new covalent C-N bond is formed and the N-glycosidic bond is broken, the negatively charged uracil base is protonated and released.

Over the years, a large number of crystal structures of UDG enzymes have been reported. The solved UDGX structures reveal a similar structural fold like other UDG enzymes with known structures. The extended loop located in motif 3 contains the critical cross-linking site (H109 in Msm UDGX) that differentiates UDGX from other conventional UDG enzymes. Besides the obvious importance of the addition of the extended loop to motif 3, several structural features in UDGX are quite distinct. The role of E52 is demonstrated by E52A mutant, which reduced the catalytic activity significantly (Figure 3 and Table 1). Structurally, the mainchain of E52 interacts with O2 of uracil in the active site, facilitating the removal of uracil in a manner similar to previous observation on family 4 UDGa and other UDG enzymes [2,4,24]. In addition, a unique interaction between E52 and the imidazole sidechain of H109 becomes feasible as H109 in the extended loop is located nearby (Figure 8A), which was also reported in previous studies [33,38]. Because the interaction is between the carboxyl sidechain of E52 and the ε^2^ nitrogen of the imidazole sidechain of H109 (the nitrogen that cross-links to the AP site), we surmise that this interaction may position H109 for the cross-linking step and may help stabilize the ε^2^ nitrogen of the imidazole and C1′ carbon of deoxyribose interaction during the transition state (Figure 9). While E52A retained partial cross-linking activity and H109A retained reduced uracil excision activity, the E52A-H109A double mutant was inactive (Table 1). The implication is that when H109A mutation is added to E52A mutation, it would further comprise the catalytic function of the latter, suggesting that the uracil excision and the cross-linking are connected. This is the first case of inter-motif interaction in UDGX.

The second case of the inter-motif interaction is illustrated by the D59–R107–D56 interaction between motif 1 and motif 3, in which D59 and D56 located in an α helix anchor R107 located in the extended loop (Figures 5D and 8E) [37]. The kinetics analysis and available structural information allow us to define the tripartite interactions. We propose that the guanidino sidechain of R107 of motif 3 and the carboxyl sidechain of D59 of motif 1 form bidentate salt bridges, and this inter-motif interaction is further supported by bidentate interactions between the sidechain of R107 and the mainchain and the sidechain of D56, respectively (Figure 5D). The salt bridges between D59 and R107 are critical to maintaining the functional structure of the enzyme, since mutations at these positions could lead to the protein unfolding (Supplementary Figure S3B). Additionally, there is probably another essential role the inter-motif tripartite interactions can play, which is to anchor the H109-containing extended loop to the proximity of the active site to ready it for cross-linking reaction.

In addition to the inter-motif interactions, the mutational and kinetics analyses also uncover an intra-motif hydrogen bond between the mainchain of N91 and the sidechain of K94 (Figure 8B). The mutation of K94 caused the loss of normal protein folding (Supplementary Figure S3B), suggesting this interaction is critical to the entire protein structure. However, the N91A mutant still maintained the protein folding (Supplementary Figure S3A), which is probably because the mainchain of the alanine could also interact with the sidechain of K94 vicariously. The first residue in motif 3 (N91 in UDGX) plays an important catalytic role in some UDG enzymes by forming bidentate hydrogen bonds with the uracil base. In *E. coli* family 2 MUG enzyme, conversion of K68 (equivalent to N91 in UDGX) to Asn leads to greatly enhanced glycosylase activity toward all mismatched T/U, G/U and C/U base pairs and acquisition of activity on A/U base pairs [6]. Msm UDGX is most homologous to family 4 UDGa such as Tth UDGa (Figure 1B). Interestingly, we found potentially a similar interaction between the mainchain of N80 and the sidechain of K83 in Tth UDGa (Figure 8C). In UDGX, the bidentate interactions of N91 with the departing uracil base and the hydrogen bond interaction with K94 are essential for catalysis.

### Functional coupling

The structural coupling described above provides a basis for discussing functional coupling detailed below. Unlike a conventional UDG, which will dissociate from the AP site generated by uracil excision or displaced by a downstream AP endonuclease during Base Excision Repair (BER). UDGX performs cross-linking reaction upon uracil excision. The discussion above on E52 and H109 already suggests that uracil excision and cross-linking are connected. A comparison of H109A mutant with the wild-type UDGX offers another clue about the coupling of uracil excision and cross-linking. As shown in Figure 6B, H109A was able to generate the AP site product, which was hydrolyzed to smaller fragments after alkaline treatment. However, we consistently failed to observe any AP site product when an uracil-containing substrate was treated with the wild-type UDGX (Figure 7A,C,D). These results suggest that once an uracil base is excised, the resulting AP site intermediate is likely immediately cross-linked by UDGX, indicating a coupling of the two catalytic steps. Lastly, we let the wild-type UDGX react with the AP site substrate but could not detect any cross-linking product (Figure 7B). These results suggest that UDGX could not cross-link to an existing AP site without coupling to a preceding uracil excision step. For all the evidence described above, we conclude that in the UDGX-catalyzed reaction, the uracil excision and cross-linking steps are tightly coupled.

### Catalytic mechanism

The catalytic mechanisms in various families of enzymes in UDG superfamily are extensively investigated [2,4,40,41]. Key catalytic residues in motifs 1, 2 and 3 have been identified and studied. As a member of the UDG superfamily, UDGX maintains a catalytic mechanism for uracil excision in a manner similar to what is proposed for its close homolog in family 4 UDGa [24]. Here, we propose a catalytic mechanism for the UDGX-catalyzed uracil excision and cross-linking to the AP site, integrating the conventional mechanism with the structural and functional coupling information described above. Similar to family 4 UDGa, the uracil base in the active site is recognized by the bidentate hydrogen bond between O3 and N4 of uracil and the N91 of motif 3, stacked by the aromatic ring of the F65 of motif 1. Likewise, the O2 of uracil is contacted by a mainchain interaction with E52 of motif 1 and the imidazole ring of H178 of motif 2. What sets UDGX apart from family 4 UDGa and other families in the UDG superfamily is the structural coupling and consequent functional coupling. The extended loop of motif 3 is brought to the proximity of the scissile bond and cross-linking site by the E52–H109 interaction and D59–R107–D56 tripartite interactions. As a consequence, the interaction between the mainchain of E52 and O2 of uracil becomes dependent on E52–H109 interaction. Likewise, the bidentate interaction between the amide sidechain of N91 and O3 and N4 of uracil relies on the support of N91–K94 interaction. These interdependent inter-motif and intra-motif interactions lead to concerted actions in catalysis. According to the results and functional coupling discussed above, the catalytic mechanism bears similarities to previously proposed models for UDGX and other UDG enzymes but with the distinct feature of coupling the two catalytic steps [33,37,38,40]. During the uracil excision step, while several interactions including E52, N91, H178 contribute to the promotion of uracil departure, the reaction is immediately coupled to the second catalytic step, in which H109 initiates the attack at C1′ carbon to form the C-N bond. The breakage of N-glycosidic bond and the departure of uracil are likely promoted by E52-O2 of uracil and H178-O2 of uracil interactions that stabilize the negative charge developed on uracil during the catalysis. Meanwhile, guided by the well knitted network of interactions, H109 is poised to initiate attack at the C1′ position of the deoxyribose to form a new covalent C-N bond and in the meantime to further push the departure of the uracil base, which therefore is proposed that the breaking of N-glycosidic bond and the forming of new covalent C-N bond are completed almost simultaneously (Figure 9, transition states).

In conclusion, a structurally coupling network is identified in UDGX, as a result, a functional coupling of uracil excision and DNA cross-linking is proved to exist. Based on the understanding of the structural and functional coupling of UDGX, a distinctive coupling catalytic mechanism is proposed, different from other enzymes in UDG superfamily. Thus, evolution has allowed UDGX to build an elegant network of interactions to enable functional coupling to achieve concerted completion of uracil excision and AP site cross-linking in a single catalytic reaction, providing an example of the interconnectivity of motifs to perform multi-facet catalytic function.

## Materials and methods

### Reagents, media and strains

All routine reagents were purchased from Sigma Chemicals (St. Louis, MO), Fisher Scientific (Suwanee, GA), VWR (Suwanee, GA), ThermoFisher Scientific (Waltham, MA) or Gel Company (San Francisco, CA) and all buffers were prepared in high-quality deionized water from a Thermo Scientific Nanopure Water System (Suwanee, GA) with a resistivity greater than 18.2 MΩ.cm. Plasmid miniprep kits and DNA gel extraction kits were purchased from New England Biolabs (Ipswich, MA) and ThermoFisher Scientific (Waltham, MA). Thermal shift protein stability kit was purchased from Biotium Inc (Fremont, CA). Restriction enzymes, Phusion DNA polymerase, T4 DNA ligase and dNTP were purchased from ThermoFisher Scientific (Waltham, MA). Cod UNG (heat-labile UDG from *Gadus morhua*) was purchased from ArcticZymes (Tromsø, Norway). HisTrap FF (1 mL), HiTrap Q FF (1 mL) and HiTrap SP FF (1 mL) columns were purchased from GE Healthcare Life Sciences (Piscataway, NJ). Hi-Di formamide and GeneScan 500 LIZ dye size standard for ABI 3130xl were purchased from Applied Biosystems. Gene strands and oligonucleotide primers for PCR were synthesized from Eurofins Genomics (Huntsville, AL). Oligodeoxynucleotide substrates with carboxyfluorescein (FAM) fluorescence label were ordered from Integrated DNA Technologies Inc. (Coralville, IA). The LB medium was prepared according to standard recipes. The sonication buffer consisted of 50 mM Tris-HCl (pH 7.5), 300 mM NaCl and 40 mM imidazole. Buffer A for HisTrap FF columns consisted of 50 mM Tris-HCl (pH 7.5), 400 mM NaCl and 10% glycerol; buffer B consisted of 20 mM Tris-HCl (pH 7.5), 400 mM NaCl, 500 mM imidazole and 10% glycerol. Buffer A for HiTrap Q/SP FF columns consisted of 50 mM Tris-HCl (pH 7.5) and buffer B consisted of 50 mM Tris-HCl (pH 7.5) and 1 M NaCl. *E. coli* strain DH5α was purchased from ThermoFisher Scientific (Waltham, MA) and *E. coli* strain Rosetta was purchased from VWR (Suwanee, GA).

### Identification of UDG genes in sequenced genomes

A tree of life based on 81 bacterial species was obtained from TimeTree [42] and visualized by iTOL [43]. UDG gene distribution of each bacterial species was superimposed into the tree of life. The amino acid sequences of UDG families and UDGX genes were obtained by searching non-redundant protein sequences (nr) database within NCBI using BLASTP [44]. For family 1 UNG, family 2 TDG/MUG, family 3 SMUG1, family 4 UDGa, family 5 UDGb, family 6 HDG and UDGX genes, *E. coli* UNG (Genbank accession number NP_289138.1), *E*. *coli* MUG (Genbank accession number P0A9H1.1), *Geobacter metallireducens* SMUG1 (Genbank accession number YP_383069.1), *Thermus thermophiles* UDGa (Genbank accession number WP_011228142.1), UDGb (Genbank accession number WP_011173217.1), *Methanosarcina barkeri* HDG (Genbank accession number WP_011305765.1), *Mycobacterium smegmatis* UDGX (GenBank accession number: WP_011726794.11) were used as a query.

### Cloning, expression and purification of UDGX

The UDGX family uracil-DNA binding protein gene (*ugdx*) from *Mycobacterium smegmatis* str. MC2 155 (GenBank accession number: CP009494.1) was amplified by PCR using the forward primer 5′-TGCATATGGCGGGTGCGCAAGAT-3′ (Nde I) and the reverse primer 5′- CAAGCTTGCAGATGGGCTCCATC-3′ (Hind III). The PCR reaction mixture (50 μl) consisted of 25 ng genomic DNA template, 100 nM forward and reverse primers, 1x Phusion DNA polymerase buffer, 200 μM each dNTP, and 0.5 Unit of Phusion DNA polymerase (ThermoFisher Scientific). The PCR procedure included a pre-denaturation step at 98°C for 1 min; 35 cycles of three-step amplification with each cycle consisting of denaturation at 98°C for 10 s, annealing at 60°C for 20 s, and extension at 72°C for 90 s; and a final extension step at 72°C for 7 min. The PCR product was purified and digested with Nde I and Hind III. After digestion, the PCR product was ligated with Nde I/Hind III digested pET28b or pET21a vector with T4 DNA ligase. The ligation mixture was then transformed into *E. coli* strain DH5α competent cells. The recombinant plasmid was finally verified by DNA sequencing with T7 promoter and T7 terminator primers.

PCR based site-directed mutagenesis was performed using the pET28b-udgx or pET21a-udgx recombinant plasmid as the template similarly as previously described [45]. The PCR reaction mixture (40 μl) consisted of 20 ng DNA template, 50 nM of each primer pair carrying the desired mutations, 200 μM each dNTP, 1x Phusion DNA polymerase buffer and 1 Unit of Phusion DNA polymerase. The PCR procedure included a pre-denaturation step at 98°C for 2 min; 25 cycles of three-step amplification with each cycle consisting of denaturation at 98°C for 10 s, annealing at 65°C for 20 s, and extension at 72°C for 7.5 min; and a final extension step at 72°C for 5 min. The PCR product was then treated with Dpn I and transformed into *E. coli* strain DH5α competent cells. Successfully mutated plasmid was confirmed by DNA sequencing. The double mutants of UDGX were generated by two rounds of PCR based site-directed mutagenesis. Taking the mutant Q53A-H109A as an example: the mutant Q53A was first generated by PCR using the pET28b-udgx recombinant plasmid as the template and the primer pair carrying Q53A mutation; then using the pET28b-udgx-Q53A plasmid as the template, the double mutant Q53A-H109A was generated by PCR with the primer pair carrying H109A mutation.

To express the wild-type and mutants of UDGX, the recombinant plasmids were transformed into *E. coli* strain Rosetta competent cells. Culture incubation, IPTG-induced protein expression and purification were performed as described below. A single *E. coli* colony transformed with recombinant plasmid was cultured in 500 mL LB medium supplemented with 50 μg/ml Kanamycin or 100 μg/ml Ampicillin at 37°C with shaking at 250 rpm until the optical density at 600 nm was over 0.6. After adding IPTG to a final concentration of 0.8 mM, the culture was grown at 22°C overnight. The *E. coli* cells were harvested at 5000 rpm with a JLA8.1000 rotor (Beckman Coulter) at 4°C for 20 min. The cell pellet was resuspended in 7 ml sonication buffer and then sonicated at 5 s on/ 5 s off cycle for 10 min on ice using Qsonica model Q125. The sonicated solution was then centrifuged at 12000 rpm with a JLA16.250 rotor (Beckman Coulter) at 4°C for 20 min. The supernatant was transferred into a fresh tube and loaded onto a 1 mL HisTrap FF column. After the sample loading, the column was washed with 15 ml of buffer A. The bound protein in the column was eluted with a linear gradient of 0–100% buffer B and collected in 1 ml fractions. The fractions identified by UV280 and SDS-PAGE were pooled and diluted 5-fold with 50 mM Tris-HCl (pH 7.5) buffer. The diluted solution was loaded on to a 1 ml HiTrap Q FF or HiTrap SP FF column. After the column was washed with 15 ml of buffer A, the bound protein in the column was eluted with a linear gradient of 10–100% buffer B and collected in 1 ml fractions. After identification, the fractions containing wild-type or mutants of UDGX were pooled and concentrated. The protein concentration was determined by Nanodrop One (ThermoFisher Scientific). The protein solution was stored at −20°C in 50% glycerol.

### Oligodeoxynucleotide substrates

Oligodeoxynucleotide substrates containing uracil (Figure 1A) were prepared as previously described^16^. Carboxyfluorescein (FAM) fluorescence-labeled single-stranded oligodeoxynucleotides containing deoxyuridine (U) and 1.5-fold molar excess complementary single-stranded oligodeoxynucleotides were mixed and incubated at 85°C for 3 min, followed by annealing to form duplex DNA substrates at room temperature for more than 30 min.

An apurinic/apyrimidinic site (AP site) containing single-stranded oligodeoxynucleotide substrate was produced by incubating 5 μM of uracil-containing single-stranded oligodeoxynucleotides with 1 unit of Cod UNG at 37°C for 30 min. After incubation, the Cod UNG was inactivated completely and irreversibly by heating at 55°C for 20 min [46].

### DNA cross-linking assay

DNA cross-linking assays for wild-type and mutants of UDGX were performed at 37°C for 30 min in 20 μl reaction mixtures containing 100 nM uracil-containing DNA substrate, 100 nM ([E]:[S] = 1:1) or 10000 nM enzyme [E]:[S] = 100:1), 25 mM Tris-HCl buffer (pH 7.5), 1 mM EDTA and 1 mM DTT. The mixtures were heated at 95°C for 5 min after adding 5 μl SDS-PAGE loading buffer, followed by loading into 15% SDS-PAGE gel. Electrophoresis was conducted at 300 V for 30 min. Fluorescence labelled oligodeoxynucleotides in the gel were visualized by a Typhoon FLA 7000 imager (GE Healthcare).

### Uracil excision assay

Uracil excision assays for UDGX-H109A, E52A-H109A and Q53A-H109A mutants were performed at 37°C for 30 min in 10 μl reaction mixtures containing 10 nM oligodeoxynucleotide substrate, 1000 nM enzyme, 25 mM Tris-HCl buffer (pH 7.5), 1 mM EDTA and 1 mM DTT. The resulting abasic sites were cleaved by heating at 95°C for 5 min after adding 1 μl of 1 M NaOH to stop the reaction. The mixtures (2 μl) were then mixed with 7.8 μl Hi-Di formamide and 0.2 μl GeneScan 500 LIZ dye size standard, and analyzed by Applied Biosystems 3130xl sequencer with a fragment analysis module. Cleavage products and remaining substrates were quantified by GeneMapper software.

### Enzyme kinetics analysis

Excess enzymes (range from 200 to 2500 nM) of wild-type and mutants of UDGX were incubated with 20 nM of A/U or G/U base pair containing double-stranded DNA substrates, 25 mM Tris-HCl buffer (pH 7.5), 1 mM EDTA and 1 mM DTT at 37°C in 10 μl reaction mixtures. Samples were collected at 10, 30 s, 1, 1.5, 2.5, 5, 7.5, 10 and 15 min for the wild-type UDGX; at 5, 10, 15, 20, 30, 40, 55, 70 and 90 min for mutants by adding 10 μl of 100 mM NaOH to terminate the reactions. After heating at 95°C for 5 min, the samples were supplemented with 5 μl of 50% glycerol loading buffer and electrophoresed at 130 V for 100 min on a 15% urea denaturing polyacrylamide gel in 0.5 × TB buffer (44.6 mM Tris base and 44.6 mM boric acid) supplemented with 2.5 mM EDTA. The intensities of the fluorescence signals of the cross-linked product and free DNA species were quantified using a Typhoon 7000 FLA imager and ImageQuant TL software (GE Healthcare).

For H109A and Q53A-H109A mutants, excess enzymes ranging from 200 to 2000 nM were incubated with A/U or G/U DNA substrates at 37°C in 5 μl reaction mixtures supplemented with 25 mM Tris-HCl buffer (pH 7.5), 1 mM EDTA and 1 mM DTT. Samples were collected at 5, 10, 15, 20, 30, 40, 55 and 70 min by adding 5 μl of 100 mM NaOH to terminate the reactions. After incubation at 95°C for 5 min, 2 μl of reaction mixtures were mixed with 7.8 μl Hi-Di formamide and 0.2 μl GeneScan 500 LIZ dye size standard. Samples were then analyzed by ABI 3130xl with a fragment analysis module. Cleavage products and remaining substrates were quantified by GeneMapper software.

The apparent rate constants (*k*_obs_) for each concentration of the wild-type and mutants of UDGX were determined by nonlinear fitting using the integrated first-order rate (eqn 1):

(1) $$\text{P}={\text{P}}_{\text{max}}(1-{\text{e}}^{-k_{\text{obs}}\text{t}})$$

where *P* is the product yield, *P*_max_ is the maximal yield, *t* is time and *k*_obs_ is the apparent rate constant.

The kinetic parameters *k*_2_ and *K*_m_ were obtained from plots of *k*_obs_ against the total enzyme concentration ([*E*_0_]) using a standard hyperbolic kinetic expression with the program GraphPad Prism 9 following the (eqn 2) [24,35,36]:

(2) $$k_{obs}=\frac{k_{2}[E_{0}]}{K_{m}+[E_{0}]}$$

For the wild-type and some mutants, because of a large *K*_m_, in which *K*_m_ »[*E*_0_], the kinetic parameter *k*_2_/*K*_m_ values were obtained from plots of *k*_obs_ against the total enzyme concentration ([*E*_0_]) using a linear regression with the program GraphPad Prism 9 following the (eqn 3) [24,35,36]:

(3) $$k_{obs}=\frac{k_{2}[E_{0}]}{K_{m}}$$

### Thermal shift protein stability assay

Thermal shift protein stability assays for UDGX wild-type and mutants were performed in 20 μl assay mixtures containing 1.0 μg/μl of proteins, 1x GloMelt™ dye (Biotium), 25 mM Tris-HCl buffer (pH 7.5), 1 mM EDTA and 1 mM DTT in a 96-well PCR plate. The plate was incubated at 25°C for 10 min in a QuantStudio 3 Real-Time PCR system (Applied Biosystems) and then gradually heated to 95°C with a heating rate of 0.05°C/s. The fluorescence signals of the dye were captured in the SYBR® Green channel every 2.2 s, and plotted with the program GraphPad Prism 9 for the melting curves.

## Supplementary Material

Supplementary Figures S1-S4

## Data Availability

All data are included in the manuscript.

## Abbreviations

AP site: apurinic/apyrimidinic site
BER: base excision repair
caC: carboxylcytosine
fC: formylcytosine
HDG: hypoxanthine-DNA glycosylase
mC: methylcytosine
MUG: mismatch-specific uracil-DNA glycosylase
SMUG: single strand selective monofunctional uracil-DNA glycosylase
TDG: thymine-DNA glycosylase
UDG: uracil-DNA glycosylase
UNG: uracil-*N*-glycosylase
