# Supplementary material for: Structural and functional coupling in cross-linking uracil-DNA glycosylase UDGX
Source: Biosci Rep. 2024 Jan 9;44(1):BSR20231551. doi: 10.1042/BSR20231551 (PMC10776899; doi:10.1042/BSR20231551)
Supplement: Supplementary Figures S1-S4 [file BSR-2023-1551_supp.pdf]

Supplementary Data for

Structural and Functional Coupling in Crosslinking Uracil-DNA Glycosylase UDGX

Chuan Liang, Ye Yang, Ping Ning, Chenyan Chang, and Weiguo Cao*

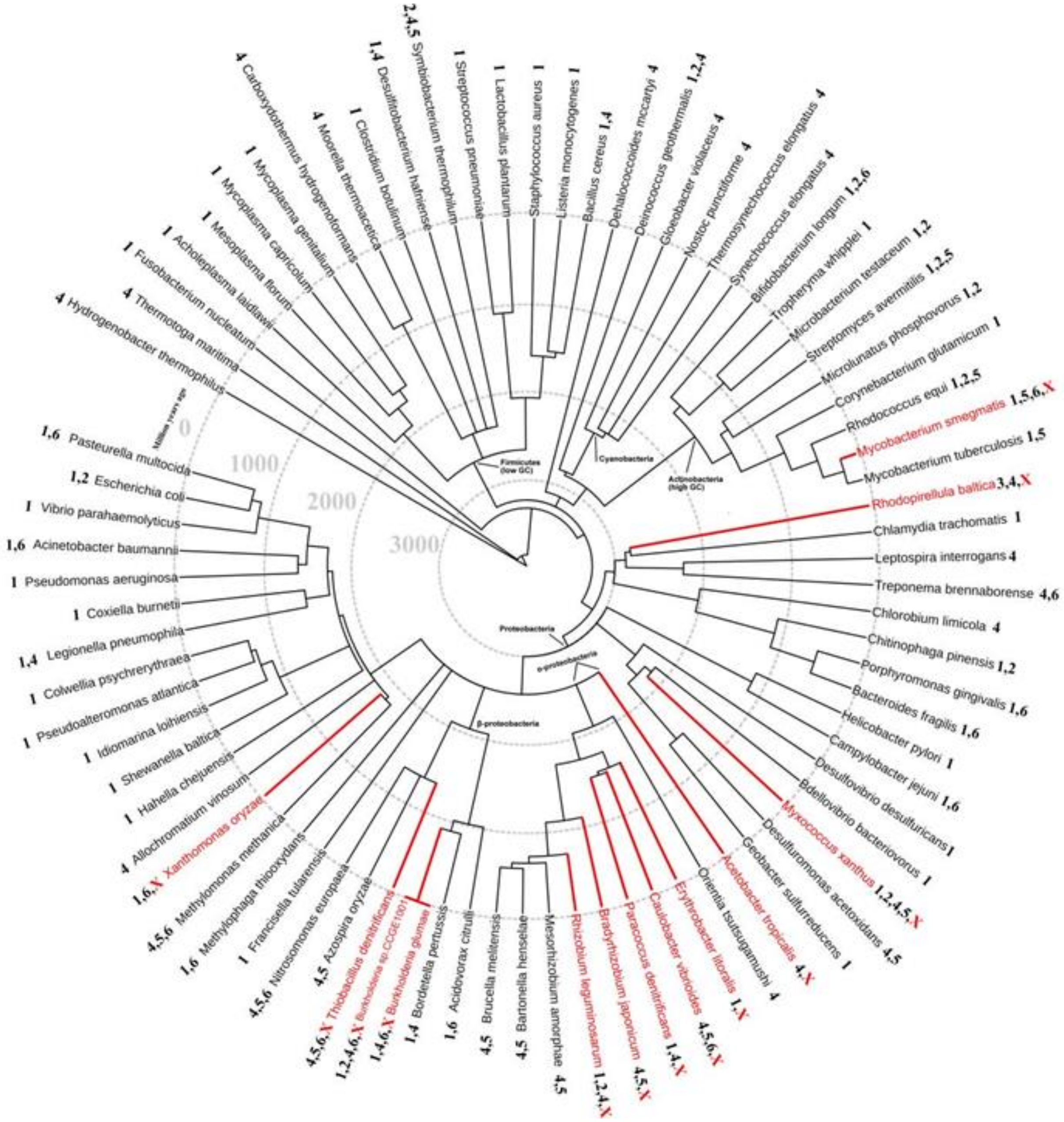

Fig. S1. Overview of UDG superfamily in selected bacterial genomes. The species tree was obtained from TimeTree and visualized by iTOL . Numbers after species names mean different UDG superfamily genes in bacterial genomes, 1, family 1 UNG; 2, family 2 TDG/MUG; 3, family 3 SMUG1; 4, family 4 UDGa; 5, family 5 UDGb; 6, family 6 HDG; and X, UDGX. Multiple genes within a family are shown as a superscript.

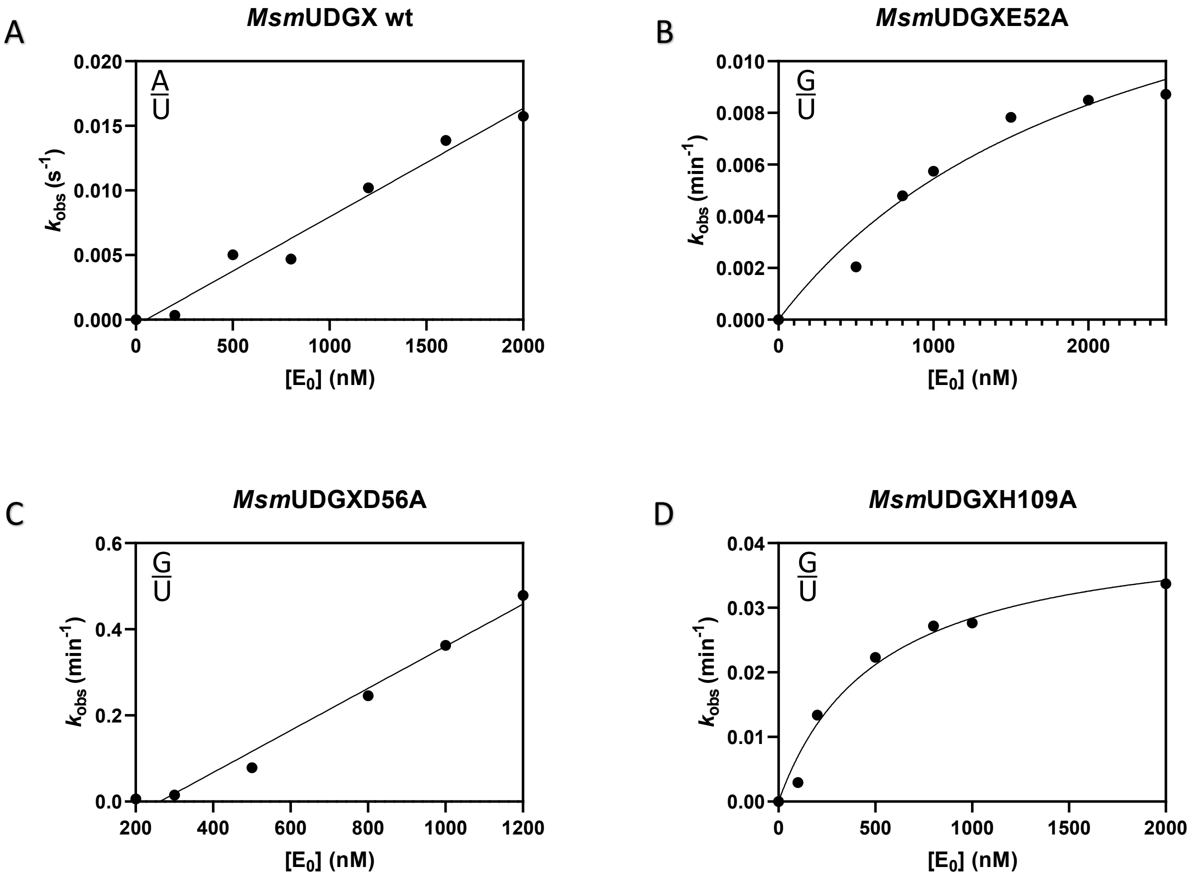

Fig. S2. Representative kinetics analysis of the wild-type and mutant UDGX. See Enzyme kinetics analysis in Material and methods for details. **A.** UDGX wild-type on A/U base pair DNA substrate. **B.** UDGX-E52A mutant on G/U base pair DNA substrate. **C.** UDGX-D56A mutant on G/U base pair DNA substrate. **D.** UDGX-H109A mutant on G/U base pair DNA substrate.

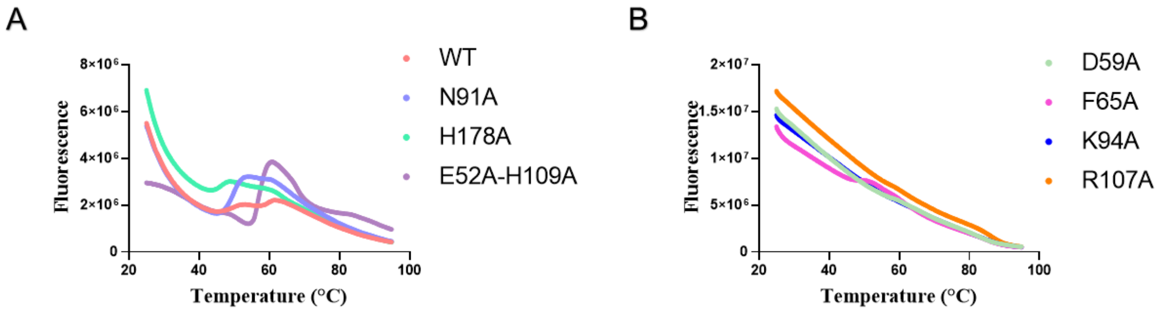

Fig. S3. Thermal shift protein stability analyses of UDGX wild-type and mutants. See Thermal shift protein stability assay in Material and methods for details. Data are shown as the average of three independent experiments. A. Fluorescence data plotting for wild-type, N91A, H178A and E52A-H109A mutants. B. Fluorescence data plotting for D59A, F65A, K94A and R107A mutants.

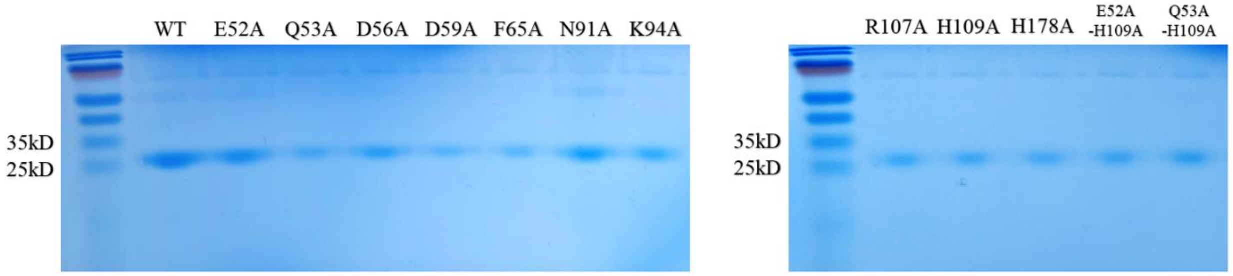

Fig. S4. SDS-PAGE gel of the wild-type and mutant UDGX. Purified proteins were electrophoresed on 10% SDS-PAGE gel, followed by Coomassie blue staining.
